# Expression Analysis Reveals That Sorghum Disease Resistance Protein SbSGT1 Is Regulated by Auxin

**DOI:** 10.3390/biology11010067

**Published:** 2022-01-02

**Authors:** Junmei Jiang, Jun Chen, Liting Luo, Lujie Wang, Hao Ouyang, Mingjian Ren, Xiangyang Li, Xin Xie

**Affiliations:** 1 State Key Laboratory Breeding Base of Green Pesticide and Agricultural Bioengineering, Key Laboratory of Green Pesticide and Agricultural Bioengineering, Ministry of Education, Guizhou University, Guiyang 550025, China; jjmguangan@163.com; 2 College of Agriculture, Guizhou University, Guiyang 550025, China; chenjun9506@163.com (J.C.); luoliting9911011@163.com (L.L.); wanglujie2806@163.com (L.W.); oyh981202@163.com (H.O.); rmj72@163.com (M.R.); 3 Guizhou Branch of National Wheat Improvement Center, Guizhou University, Guiyang 550025, China

**Keywords:** SbSGT1, auxin, interaction, expression, plant hormones

## Abstract

**Simple Summary:**

SGT1 (suppressor of the skp1 G2 allele, a component of the ubiquitin ligase complex), which is an important plant disease resistance-related protein, acts as a regulatory factor for signal transduction during the process of plant disease resistance, and is an important regulatory factor of defense response signals. Plant immunity is mediated by hormones, such as auxin; however, the molecular relationship between auxin and SGT1 remains unclear. In this study, we determined the expression patterns of *SbSGT1* under different plant hormone treatments and the result showed that *SbSGT1* was significantly upregulated. Thus, we examined the molecular interaction of SbSGT1 and indole-3-acetic acid in vitro and in vivo. The results will provide a reference for further investigation of the mechanisms underlying SbSGT1 involvement in the auxin signal transduction pathway.

**Abstract:**

SGT1 (suppressor of the skp1 G2 allele) is an important plant disease resistance-related protein, which plays an important role in plant resistance to pathogens and regulates signal transduction during the process of plant disease resistance. In this study, we analyzed the expression profile of *SbSGT1* in sorghum under phytohormones treatment. Quantitative real-time PCR results showed that *SbSGT1* was most expressed in sorghum leaves, and could respond to plant hormones such as auxin, abscisic acid, salicylic acid, and brassinolide. Subsequently, we determined the optimal soluble prokaryotic expression conditions for SbSGT1 and purified it using a protein purification system in order to evaluate its potential interactions with plant hormones. Microscale thermophoretic analysis showed that SbSGT1 exhibited significant interactions with indole-3-acetic acid (IAA), with a Kd value of 1.5934. Furthermore, the transient expression of SbSGT1 in *Nicotiana benthamiana* indicated that treatment with exogenous auxin could inhibit SbSGT1 expression, both at the transcriptional and translational level, demonstrating that there exists an interaction between SbSGT1 and auxin.

## 1. Introduction

The levels of hormones, such as auxin, salicylic acid (SA), jasmonate acid (JA), abscisic acid (ABA), brassinosteroids (BR), and ethylene (ET), either increase or decrease to regulate gene expression when plants are faced with the abiotic or biotic stresses [1]. However, the mechanism of auxin regulation is different from that of other plant hormones. When auxin is applied externally, it can enhance plant susceptibility to pathogens; for example, auxin can increase the susceptibility of rice to *Magnaporthe oryzae* and rice bacterial leaf blight [2,3].

The auxin signaling pathway is involved in the regulation of plant physiological and biochemical processes. The protein components and genes related to the auxin signal transduction pathway mainly include the SCF (SKP1-CDC53/CUL1-F-box) complex, the auxin response factor, and SGT1. Indole-3-acetic acid (IAA) can regulate the entire plant growth and development process [4]. For example, it is involved in plant tissue differentiation and response to different environmental stress signals. In addition, the auxin signal transduction pathway, which is relatively complex, is composed mainly of three phases, which are, transduction signal recognition, downstream gene expression, and plant phenotype appearance. Reports have shown that the main proteins involved in auxin signal transduction pathway are auxin/indoleacetic acid proteins (Aux/IAAs), SKP1-CDC53/CUL1-F-box (SCF), KRP2 (Kip-related protein 2), E2Fa transcription factor, auxin binding protein 1 (ABP1), PROPORZ1(PRZ1), and the auxin response factor (ARF) [5,6].

*SGT1* (suppressor of the skp1 G2 allele), which is an important plant disease resistance-related gene, acts as a regulatory gene for signal transduction during the process of plant disease resistance, and is an important regulatory factor of defense response signals. *SGT1* gene was first discovered in yeast. It regulates centromere assembly and genes related to ubiquitination [7]. This gene was subsequently identified in plants and animals [8]. The amino acid sequence of the SGT1 protein in plants contains five domains, i.e., a tetratricopeptide repeat at the N-terminal repeat structure (TPR), variable regions 1 (VR1) and 2 (VR2), a CHORD and SGT1 motif (CS), and an SGT1-special group at the C-terminal motif (SGS) [9]. It has been reported that the TPR and CS regions of the repeat structure at the N-terminus, the CS region, and the SGS region can interact with the HSP90 protein, the RAR1 protein, and the LRR region of the MLA1 protein, respectively [10,11]. During protein ubiquitination, SGT1 usually interacts with the SCF complex to regulate the ubiquitination and degradation of Aux/IAAs, resulting in the ARF, a transcription activator and a downstream gene expression stimulant in the auxin transduction signal pathway. In addition, OsCYP2 interacts with OsSGT1 during the process of auxin signal transduction in rice [12]. *SGT1*, as a disease resistance-related gene, confers a certain level of resistance to plants against diseases caused by bacteria, fungi, viruses, and other pathogens, indicating that it may participate in the plant disease resistance signaling pathway [13,14].

In this study, we evaluated the changes of *SbSGT1* expression in sorghum seedlings at different time points after treatment with plant hormones. In addition, we analyzed interactions between SbSGT1 and plant hormones based on micro scale thermophoresis (MST) technology and measured the expression levels of SbSGT1 after auxin treatment in vivo. This study provides a reference for further investigation of the mechanism of SbSGT1 involvement in the auxin signal transduction pathway.

## 2. Materials and Methods

### 2.1. Plant Growth Conditions and Treatments

Sorghum seeds were sanitized with 40% sodium hypochlorite, soaked in clean water for 24 h, and moisturized with filter paper for 48 h. The germinated seeds (25 °C) were planted in sterilized nutrient soil (PINDSTRUP, Denmark) and cultivated in a greenhouse with a relative humidity of 75% at 25/20 °C under a 14 h light/10 h dark cycle. In the three-leaf stage, the roots, stems, leaves, and buds of the seedlings were collected for tissue specific analysis. For plant hormone treatment, the three-leaf stage leaves were sprayed by using ABA (200 µM), IAA (10 µM), SA (1 mM), BR (1 µM), and strigolactone (SL) (1 µM) separately. Then, all samples were obtained at 0, 0.5, 1, 3, 6, 9, 12, and 24 h post treatment, and then biological replicates were set up. Five plants were treated in each biological replicate. The samples were quickly frozen in liquid nitrogen and stored in a freezer at −80 °C for RNA extraction.

### 2.2. Prokaryotic and Transient Expression Plasmid Construction

RNA was extracted from sorghum seedlings using Trizol, and cDNA was synthesized using the cDNA Synthesis Kit. To construct the prokaryotic and transient expression plasmids, the primer pair, *SbSGT1*-F/*SbSGT1*-R was used to amplify *SbSGT1* from the cDNA template (Table A1). The amplification system was composed of 14.5 µL of buffer, 5.5 µL of ddH_2_O, 1.5 µL of dNTP, 1 µL of each primer, 1 µL of cDNA template, and 0.5 µL of KOD high-fidelity enzyme. The PCR program were set as follows: 32 cycles of 98 °C for 1 min, 98 °C for 15 s, and 68 °C for 1 min. PCR products were examined by 1% agarose gel electrophoresis and the enzymes, *EcoR* I and *Xho* I, were subsequently used to digest the PCR products, and pET-28a (prokaryotic expression vector) and pYBA-1143 (transient expression vector), respectively. After ligation, the products were transferred to *Escherichia coli* (*E. coli*) DH5*α* using the heat shock method (42 °C, 45 s), and incubated overnight at 37 °C. Single colonies were confirmed by sequencing.

### 2.3. Analysis of SbSGT1 Promoter Cis-Acting Elements

The 2000 bp upstream sequence of *SbSGT1* was downloaded from the sorghum database (https://phytozome.jgi.doe.gov/pz/portal.html 10 September 2021), and the PlantCARE (http://bioinformatics.psb.ugent.be/webtools/plantcare/html/ 10 September 2021) online website was used to predict the cis-acting elements in the *SbSGT1* promoter.

### 2.4. SbSGT1 Expression Profiles

Real-time fluorescent quantitative PCR (qPCR) was used to determine the expression patterns of *SbSGT1*. The amplification system was composed of 4.5 µL of cDNA, 7.5 µL of SYBR mix, 0.3 µL of *SbSGT1*-qF and *SbSGT1*-qR, and 15 µL of ddH_2_O. The PCR program were set as follows: One cycle at 95 °C for 5 min, 40 cycles at 95 °C for 10 s, 58 °C for 30 s, 72 °C for 30 s. Three biological replicates were set up, and *SbEIF4a* (eukaryotic translation initiation factor 4a) was used as the internal reference gene. qPCR data were analyzed using the 2^−ΔΔCt^ method, and SPSS was employed for significance examination.

### 2.5. Soluble Expression Conditions Screening for SbSGT1

The recombinant plasmid, pET-28a-SbSGT1 was transformed into the *E. coli* expression strains, BL21 (DE3), JM109 (DE3), and Rosetta (DE3), using the heat shock method, and gene expression was induced using 0.8 mM IPTG at 25 °C for 10 h. We collected 6 mL of bacterial solution, centrifuged it at 12,000 rpm for 2 min, and added to it 400 μL of lysate buffer (50 mM Tris pH 7.5, 1 mM EDTA, 100 mM NaCl, 1 mg/mL lysozyme, 1% Triton X-100, and protease inhibitor). The cells were disrupted with an ultrasonic disruptor, centrifuged at 12,000 rpm for 10 min, and the supernatant and pellets were separated, boiled for 5 min, and centrifuged at 12,000 rpm for 2 min. To determine the most effective expression strain, the supernatant was collected and electrophoresed by 12% SDS-PAGE to determine the expression levels of the target protein. The different protein expression conditions, including the induction temperatures (16, 20, 25, 30, and 37 °C) and Isopropyl-*β*-D-thiogalactopyranoside (IPTG) concentrations (0, 0.2, 0.4, 0.6, 0.8, 1.0, and 1.2 mM) were determined.

### 2.6. SbSGT1 Purification and Desalting

A large quantity of the bacteria expressed SbSGT1 protein was collected at 4 °C, and a lysis buffer (30 mM Tris-HCl pH 8.0, 300 mM NaCl, 10% glycerol, 1% mercaptoethanol) was added to the bacterial pellets. The cells were then ultrasonicated for 30 min at 35% power, and centrifuged at 12,000 rpm for 30 min at 4 °C. The supernatant and precipitate were separated. Buffer A (20 mM imidazole, 30 mM Tris-HCl pH 8.0, 300 mM NaCl, 10% glycerol) and buffer B (350 mM imidazole, 300 mM Tris-HCl pH 8.0, 300 mM NaCl, 10% glycerol) were used to equilibrate the HisTrap^TM^ protein purification nickel column. The supernatant was passed through a peristaltic pump at 2 mL/min for a single cycle to affinity the protein 2–3 times, and the AKTA^TM^ pure protein purification system was used to wash the protein in the nickel column into buffer B; the eluate (purified protein) was collected and analyzed through 12% SDS-PAGE. Furthermore, the supernatant was filtrated through a 0.22 μm membrane, and the eluate was passed through a HisTrap^TM^ desalting column and washed with SEC buffer (300 mM Tris-HCl pH 8.0, 300 mM NaCl). Finally, the corresponding eluate was collected, and subjected to 12% SDS-PAGE to detect a purified desalted protein.

### 2.7. Micro Scale Thermophoresis (MST)

MST was carried out using Nanomper Monolith NT.115 (Germany). First, the Monolith NT™ protein labeling kit (Germany) was used to label purified protein. The equal volume of SEC buffer was added to the PCR tube, and 10 µL of 2 mM candidate substance was added to the first PCR tube. Then, 10 µL of the sample in PCR tube 1 was transferred PCR tube 2, and so on, until PCR tube 16. After, 10 µL of the labeled protein were added to the 16 PCR tubes and mixed gently. The samples were incubated in the dark at room temperature for 5 min. Then, capillary tubes were filled with the samples, and Kd values were calculated using the NanoTemper mass-effect equation. Data were expressed as mean ± SD of 3 replicates.

### 2.8. Tobacco Transient Expression, Plant Protein Extraction, and Western Blot Detection

After the tobacco seeds were disinfected using 40% sodium hypochlorite, they were sown and moisturized in nutrient soil until reached the two-leaf stage. Then, the seedlings were transplanted in a greenhouse at 22 °C for 20 days and injected with recombinant *Agrobacterium tumefaciens* stains harboring *SbSGT1*. In brief, A. tumefaciens GV3101 competent cells were transformed with recombinant plasmids by electroporation using a MicroPulser^TM^ and cultured at 28 °C for 2 days. Bacterial pellets were collected after centrifugation at 4000 rpm for 10 min; a suspension solution (10 mM MES pH 5.6, 200 μM acetosyringone, 10 mM MgCl_2_) was added to the pellets to adjust the concentration to an OD600 of 1.2. After mixing this solution with an equal volume of the P19 suspension (OD600 = 1.2), the mixture was incubated in the dark at room temperature for 3 h. Finally, a disposable syringe was used to infiltrate this mixture into tobacco leaves. IAA (0, 0.5, and 2 μM) was sprayed on tobacco leaves 24 h after the injection. Tobacco leaf samples were collected 72 h after the *A. tumefaciens* injection.

To extract proteins from tobacco, the leaves were placed in a pre-cooled mortar and were immediately ground to powder in liquid nitrogen; 0.2 mg of the powder was transferred into a pre-cooled 2.0 mL centrifuge tube, and 400 µL of plant protein extraction buffer (50 mM Tris pH 7.5, 150 mM NaCl, 0.1% NP-40, 1 mM PMSF, 4 M urea, protease inhibitor) were added to it. This mixture was placed on ice for 20 min, centrifuged at 12,000 rpm for 20 min at 4 °C, and the supernatant was transferred into a new tube and were stored at −20 °C.

Western blotting was performed using the wet-transfer method. The purified proteins were subjected to polyacrylamide gel electrophoresis, transferred to a PVDF membrane at 250 mA for 90 min, and blocked with 5% skimmed milk at room temperature for 2 h. Anti-HA primary antibody was added to the membrane, and incubated at room temperature for 2 h. Then, the membrane was washed 4 times with 1 × PBST buffer, and secondary goat anti-mouse antibody was added and incubated at room temperature for 1 h, after washed 4 times with 1 × PBST buffer, an HRP chemiluminescence substrate solution was added and protein signals were detected using a chemiluminescence detector (Tanon 5200).

## 3. Results

### 3.1. Sorghum SbSGT1 Cloning and Plasmid Construction

The *SbSGT1* gene was amplified by PCR using the *SbSGT1*-F and *SbSGT1*-R primers. The electrophoresis results showed that the PCR product was 1095 bp in length (Figure S1A). The amplification products and the prokaryotic expression vector, pET-28a, were digested using the restriction enzymes, *EcoR* I and *Xho* I, and then purified and ligated into pET-28a. Then, the recombinant plasmid pET-28a-*SbSGT1* was further examined by double digestion with *EcoR* I and *Xho* I, yielding a 1095 bp band (Figure S1B), confirming the successful insertion of *SbSGT1* into the pET-28a vector. To construct the plant expression plasmid, the amplified *SbSGT1* and the plant transient expression vector pYBA-1143 were digested using the *EcoR* I and *Xho* I, and ligated in frame. The recombinant construct, pYBA-1143-*SbSGT1*, was identified by colony PCR (Figure S1C) and double digestion (Figure S1D).

### 3.2. Putative Cis-Acting Regulatory Elements of SbSGT1

The *SbSGT1* gene promoter region contains a large number of stress-related cis-elements. These cis-acting elements can interact with transcription factors to regulate important functions. To identify the cis-acting regulatory elements of the *SbSGT1* gene, the 2 000 bp upstream *SbSGT1* promoter sequence was obtained from the sorghum genome database, and its cis-acting elements were predicted and analyzed. As shown in Table A2, there were many CAAT-box (34) and TATA-box (41) core response elements, and cis-acting elements related to gibberellin and auxin response elements, and other hormone response elements in *SbSGT1* promoter region. Therefore, we speculated that sorghum *SbSGT1* may be involved in the response to abiotic stress, indicating that it has diverse functions.

### 3.3. SbSGT1 Expression Profile

#### 3.3.1. Tissue Specificity Analysis of *SbSGT1*

**Figure 1:**
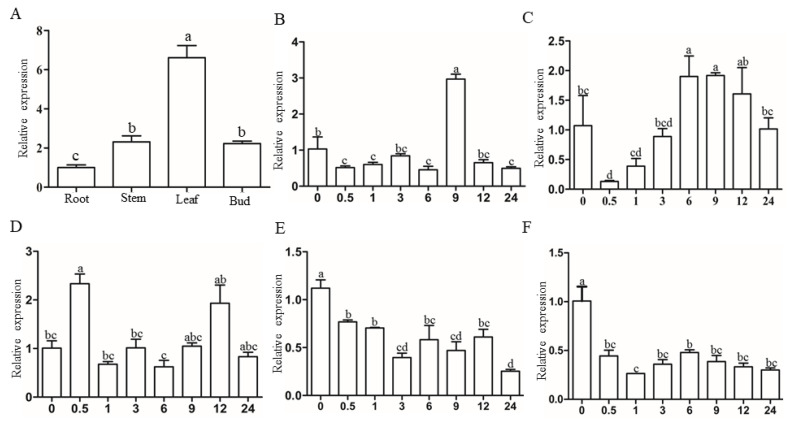
Expression profiling of *SbSGT1*. (**A**) Tissue specificity of *SbSGT1* in sorghum root, stem, leaf, and bud, respectively; (**B**–**F**) Analysis of *SbSGT1* response to indole-3-acetic (IAA), salicylic acid (SA), abscisic acid (ABA), brassinosteroids (BR), and strigolactone (SL) stresses at 0–24 h, respectively. Internal reference gene: *SbEIF4a*; Different lowercase letters indicate the significance of the difference between the treatment groups (*p* < 0.05).

To determine the expression pattern of *SbSGT1* in sorghum tissues, gene expression level from sorghum roots, stems, leaves, buds, and seeds were evaluated by qPCR using *SbSGT1*-specific primers (Table A1). As shown in Figure 1A, the expression level of *SbSGT1* in sorghum leaves was significantly higher than those in the roots, stems, and buds, being approximately six and three times higher than in the roots, and stems and buds, respectively; *SbSGT1* expression levels in stems and buds were significantly higher than those in the roots. These findings provided a basis for subsequent experiments, i.e., analysis of the effects of abiotic and biotic stress factors on *SbSGT1* expression levels in sorghum leaves.

#### 3.3.2. Analysis of Sorghum *SbSGT1* Response to Plant Hormone Stress

As shown in Table A2, the *SbSGT1* promoter region was rich in hormone response elements, especially those of auxin and ABA. To explore the response characteristics of sorghum *SbSGT1* under plant hormones, we further analyzed the expression patterns of *SbSGT1* under IAA, SA, ABA, BR, and SL stresses, and found that it was upregulated in response to these hormones.

Under IAA treatment, *SbSGT1* relative expression levels initially declined and then increased. Its relative expression levels increased after 9 h of treatment, reaching its highest levels, which were approximately three times higher than those at 0 h, at this time point, after which, its expression levels decreased again. Aside from the highest relative expression levels observed at 9 h, relative expression levels at other time points were lower than those at 0 h, indicating that auxin can promote *SbSGT1* expression within a given period (Figure 1B). After treatment with SA, *SbSGT1* was found to respond to SA, as its relative expression levels initially decreased, then increased, and decreased again. *SbSGT1* relative expression levels were lowest at 0.5 h, then gradually increased, attaining the highest levels at 9 h, and then gradually decreased (Figure 1C). Sorghum *SbSGT1* was found to respond ABA stress, as its relative expression levels showed an upward and downward trend between 0–9 h. Its expression levels were highest at 0.5 h. Between 9–24 h, its relative expression levels initially increased, reaching its highest levels at 12 h, and then decreased (Figure 1D). Under BR treatment, the relative expression levels of *SbSGT1* showed a general downward trend within the 0.5–24 h time period as compared to its levels at 0 h, with the lowest expression level being observed at 24 h (Figure 1E). After treatment with SL, *SbSGT1* was found to respond to strigolactone stress, as its relative expression showed a general downward trend, similar to its expression pattern under BR stress. *SbSGT1* expression levels between 1–24 h were low (Figure 1F).

These results indicated that sorghum could respond to stress induced by five plant hormones, including auxin, salicylic acid, abscisic acid, brassinolide, and strigolactone, and among these five plant hormone treatments, the expression level of *SbSGT1* under auxin stress was the most significant.

### 3.4. Prokaryotic Expression of SbSGT1

#### 3.4.1. Optimal Expression Strain for SbSGT1

Prokaryotic and eukaryotic expression systems can be used to express exogenous protein, and the *E. coli* prokaryotic expression system is the most used with the characteristic of suitability for large protein expression, and rapid bacteria reproduction [15,16]. However, the outcomes of the process of protein prokaryotic expression induction can be affected by bacterial expression strain, induction temperature, and IPTG concentration. Thus, the recombinant plasmid, pET-28a-SbSGT1, was transformed into BL21 (DE3), Rosetta (DE3), and JM109 (DE3) expression strains, and cultured at 37 °C. A single colony was selected for small-scale induction and expression using 0.8 mM IPTG at 25 °C for 10 h, and the resulting proteins were detected by 12% SDS-PAGE. We found a significant band at 44 kDa and the protein size was within the expected range (Figure S2). Rosetta (DE3) and JM109 (DE3) can both be expressed in the form of inclusion bodies (Figure S2A, lanes 1, 3, and 5). However, only small quantities of the SbSGT1 soluble protein were expressed in the JM109 (DE3) and Rosetta (DE3) strains (Figure S2A, lanes 2 and 4), whereas large quantities were expressed in BL21 (DE3) strains (Figure S2A, lane 6); thus, the BL21 (DE3) strain was selected for further study.

#### 3.4.2. Optimum Expression Temperature for SbSGT1

The pET-28a-SbSGT1 recombinant plasmid was transformed into the BL21 (DE3). Under the same experimental conditions, with increase in induction temperature, the expression levels of the SbSGT1 protein in the supernatant initially increased and then decreased, with the highest SbSGT1 protein expression levels observed at 25 °C (Figure S2B, lane 3). Thus, 25 °C was retained as the induction temperature for further study.

#### 3.4.3. Optimum IPTG Concentration for SbSGT1

The recombinant plasmid, pET-28a-SbSGT1, was transformed into the BL21 (DE3), and the effects of IPTG concentration on the expression levels of the SbSGT1 were evaluated at an induction temperature of 25 °C. As shown in Figure S2C, IPTG induced an increase the expression levels of the SbSGT1; however, this effect was not concentration-dependent.

#### 3.4.4. Western Blot Detection of SbSGT1

To further evaluate the expressed SbSGT1, Western blotting was used to detect the recombinant protein. As shown in Figure S3, when anti-His antibody was used to detect the induced protein, a protein band of approximately 44 kDa was observed, indicating that His-SbSGT1 protein expression induction was successful.

### 3.5. SbSGT1 Purification and Desalting

#### 3.5.1. SbSGT1 Purification

To obtain the purified SbSGT1, it was expressed and purified in large quantities by affinity chromatography. As shown in Figure S4, the SbSGT1 was successfully expressed in large quantities, mainly in the supernatant (Figure S4B, Lane 2). The Figure S4A showed that SbSGT1 was eluted from the Ni column, with the UV absorption peak of approximately 1700 units by using an AKTA protein purification system. As shown in Figure S4B, a 44 kDa band was observed, which was similar to the predicted size of the SbSGT1 protein, indicating that the SbSGT1 was eluted into the imidazole salt solution.

#### 3.5.2. SbSGT1 Desalting

The salt solution containing the SbSGT1 protein in purification was eluted from the imidazole salt solution using a desalting column. As shown in Figure S5A, when the SbSGT1 protein was eluted into the SEC buffer using the desalting column, the UV absorption peak was found to be approximately 1200 with a single peak; sampling and 12% SDS-PAGE were performed on the single peak, and as shown in Figure S5B, a significant band was observed at approximately 44 kDa, which is similar to the predicted size of the SbSGT1 protein, indicating that SbSGT1 was successfully purified and could be used for protein-molecule interaction analyses.

### 3.6. Interaction between the SbSGT1 Protein and Plant Hormones

**Figure 2:**
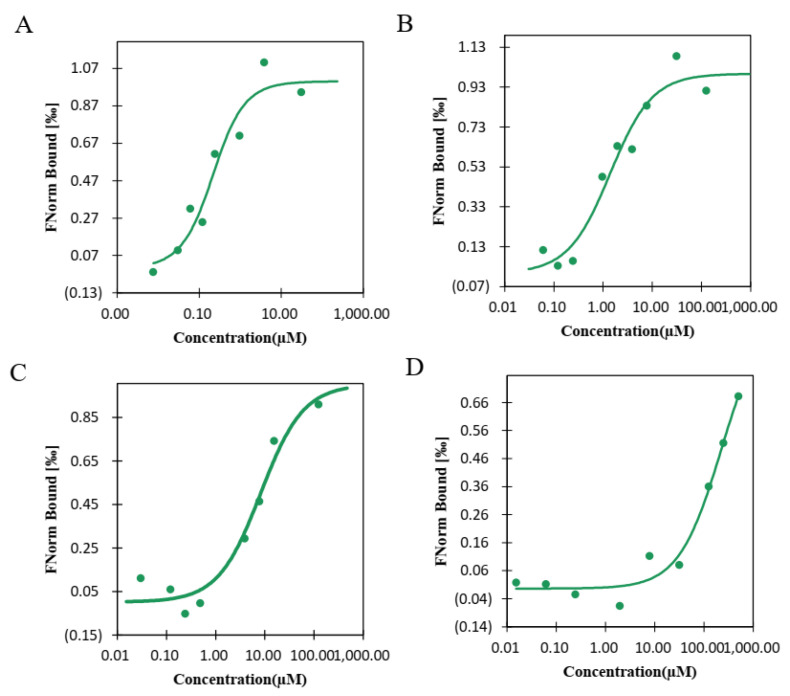
MST analysis of the interactions between SbSGT1 and plant hormones. (**A**–**C**): MST analysis of the interactions between SbSGT1 and IAA, SA, and ABA, respectively. (**D**): MST analysis of the interactions between BSA (bovine serum albumin) and IAA.

Microscale thermophoresis (MST) is a new, rapid, and accurate method for the quantitative analysis of protein-protein interaction at a microscale level. The emergence of MST, as a technique for the determination of the dissociation constants of bimolecular interactions, made it possible to measure these quantities in systems in which this was previously difficult or infeasible [17,18]. MST technology is also used in vitro to detect interactions between small molecular substances and proteins [19,20]. For example, MST was used to study the interactions between new compounds and CMV CP, and it was found that the strength of the interactions between compounds N12 and N16 and CMV CP was greater than that between other compounds and CMV CP, providing a reference for the research and development of anti-CMV viral agents [21]. In this study, we used MST to determine the interactions between the SbSGT1 protein and plant hormones, such as auxin, salicylic acid, and abscisic acid. We found that the SbSGT1 protein could be linearly fitted with IAA, SA, and ABA (Figure 2), with kD values of 1.5934, 3.3116, and 5.38, respectively (kD values varied in the order, ABA > SA > IAA). These results indicated that IAA exhibited stronger interaction with the SbSGT1 protein than the other plant hormones (Figure 2A); The interaction between BSA (bovine serum albumin) and IAA was used as the negative control), with kD values of 228.81 (Figure 2D); thus, IAA was selected for the subsequent in vivo verification of candidate plant hormones.

### 3.7. Auxin Inhibited SbSGT1 Expression at the Transcriptional and Translational Level In Vivo

**Figure 3:**
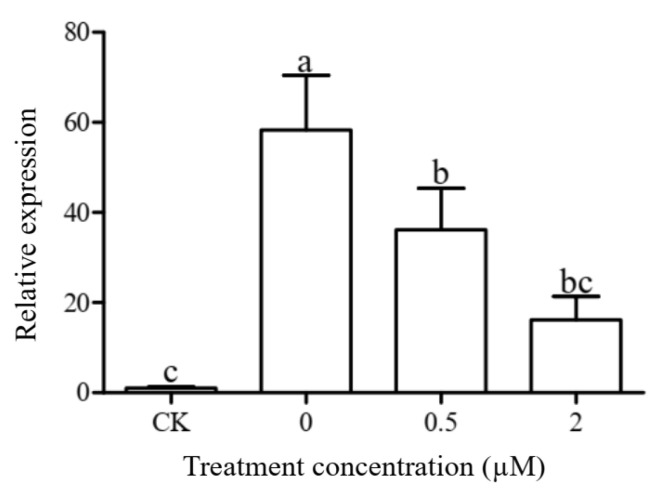
Effects of auxin on *SbSGT1* expression at the transcriptional level in *Nicotiana benthamiana*. *NbEF1*: Internal reference gene; Different lowercase letters indicate the significance of the difference in variance between the treatment groups (*p* < 0.05).

To further investigate whether auxin inhibits SbSGT1 expression in vivo, we measured SbSGT1 transcriptional and translational levels in *Nicotiana benthamiana*. As shown in Figure 3, IAA could regulate the relative expression of *SbSGT1* at the transcriptional level. Within the auxin concentration range of 0–2 μM, as its concentration increased, the relative expression of *SbSGT1* gradually decreased. The relative expression levels of *SbSGT1* at an auxin concentration of 0 μM were significantly higher than those at auxin concentrations of 0.5 μM and 2 μM. Within a given auxin concentration range, the expression levels of *SbSGT1* at the transcriptional level decreased.

**Figure 4:**
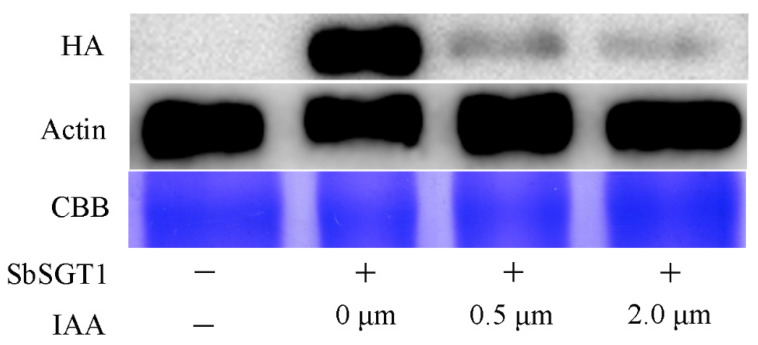
Effects of auxin on the accumulation of SbSGT1 protein level. HA: Specific antibody for the detection of SbSGT1 protein in *Nicotiana benthamiana*; Actin: plant reference protein; CBB: Coomassie brilliant blue staining; −: empty control; +: spray IAA with 0–2.0 μM after *Agrobacterium tumefaciens* harboring pYBA-1143-SbSGT1 infiltration in *N. benthamiana*.

As shown in Figure 4, auxin could equally affect SbSGT1 expression at the protein level, i.e., within an auxin concentration range of 0–2 μM. Using the specific HA antibody to detect the SbSGT1 expression, we found that as auxin concentration increased, the band became weaker, indicating that a certain auxin concentration could inhibit SbSGT1 expression at the protein level. These results indicated that after treatment with auxin, SbSGT1 expression at both the transcriptional and translational level decreased, showing that there could exist an interaction between SbSGT1 and auxin in vivo.

## 4. Discussion

Sorghum plants are subjected to various abiotic and biotic stresses during their growth period. Studies have shown that in *Capsicum annuum*, *Haynaldia villosa*, and *Brassica oleracea, SGT1* is expressed in different tissues and under various stress conditions [22,23,24]. Reports have shown that drivers of signaling pathways regulated by plant hormones, such as auxin, SA, methyl jasmonate, and ethylene, can affect plant disease resistance, for example, the plant hormones, methyl jasmonate, and ethylene, can promote the upregulation of the expression of the *Cucurbita moschata CmSGT1* gene; in addition, the expression of the cabbage gene, *BolSGT1,* was significantly altered following treatment with exogenous ABA [24,25]. These indicated that *SGT1* expression in plants may be regulated by plant hormones. Our results showed that *SbSGT1* expression was significantly altered following treatment with auxin, salicylic acid, abscisic acid, and brassinolide, further confirming the notion that *SbSGT1* expression in sorghum plants is easily affected by plant hormones.

The auxin signaling pathway is involved in the regulation of several plant physiological and biochemical processes, and the main protein components and genes related to this pathway include the SCF (SKP1-CDC53/CUL1-F-box) complex, auxin/indoleacetic acid proteins, the auxin response factor, and SGT1 [6]. Studies in tobacco and barley have shown that the SGT1 and SCF complexes form complex interactions [26]. Studies have shown that high auxin concentrations inhibit growth in plants, i.e., it can promote the shearing of the membrane protein, TMK1 (transmembrane kinase 1), and the interaction with IAA32 and IAA34 of the auxin/indole-3-acetic acid (Aux/IAA) family will enhance protein phosphorylation and stability, thereby interacting with the auxin response factor, ARF, and inhibiting its activity, and thus preventing the expression of downstream genes [5]. We found that SbSGT1 interacts with plant hormones in vitro, i.e., abscisic acid, auxin, and salicylic acid. However, as compared to the other plant hormones, SbSGT1 interacted more strongly with auxin. In vivo experiments showed that SbSGT1 expression decreased at both the transcriptional and translational level following auxin treatment.

## 5. Conclusions

Through the analysis of the cis-acting elements of the sorghum disease resistance-related gene, *SbSGT1*, we determined the expression patterns of *SbSGT1* under different ABA (200 µM), IAA (10 µM), SA (1 mM), BR (1 µM), and SL (1 µM) treatments. In addition, the optimum prokaryotic expression conditions for SbSGT1 is the BL21 (DE3) strain at 25 °C. Subsequently, we purified the SbSGT1 protein using the AKTA^TM^ protein purification system and determined the interactions between the SbSGT1 and IAA, SA, and ABA, respectively, and found that the SbSGT1 protein could interact with auxin. Furthermore, the findings of the in vivo experiments showed that auxin could inhibit SbSGT1 expression both at the transcriptional and translational level. This study provides a reference for further investigation of the mechanisms underlying SbSGT1 involvement in the auxin signal transduction pathway.

## Data Availability

Not applicable.

## Supplementary Materials

The following are available online at https://www.mdpi.com/article/10.3390/biology11010067/s1, Figure S1: Full-length amplification of the *SbSGT1* and double digestion of the recombinant plasmids. A and C: Amplification of *SbSGT1*, lane 1–2: *SbSGT1*; B: Double digestion of pET-28a-*SbSGT1* by *EcoR* I and *Xho* I; D: Double digestion of pYBA-1143-*SbSGT1* by *EcoR* I and *Xho* I; M: DNA Marker. Figure S2: Optimize protein expression conditions of SbSGT1. A: Protein expression analysis of SbSGT1 in different strains. Lane 1–6: The lysate precipitation of Rosetta (DE3), the supernatant of Rosetta (DE3), the lysate precipitation of JM109 (DE3), the supernatant of JM109 (DE3), the lysate precipitation of BL21 (DE3), the supernatant of BL21 (DE3); B: Protein expression analysis of SbSGT1 in different temperatures. Lane 1–5: Supernatant obtained from SbSGT1 culture after induction at 16, 20, 25, 30, and 37 °C, respectively; Lane 6: the lysate precipitation of SbSGT1 was induced at 25 °C; C: Protein expression analysis of SbSGT1 in different IPTG concentrations. Lane 1–7: Supernatants obtained from SbSGT1 culture after IPTG induction at 0, 0.2, 0.4, 0.6, 0.8, 1.0 and 1.2 mM, respectively. M: Protein Marker. Figure S3: Protein determination of SbSGT1 by western blot. Protein detection of SbSGT1 in Escherichia coli by anti His antibody. Lane 1 and 2, Protein from E. coli harboring recombinant pET-28a-SbSGT1 and empty vector pET-28a, respectively. Figure S4: Protein purification of SbSGT1. A: Purification and analysis of the SbSGT1 protein using the Ni column; B: SbSGT1 protein purification by 12% SDS-PAGE electrophoresis. Lane 1–5 represents the purified solutions obtained from the protein purification system. M represents the protein standard molecular weight. Figure S5: Protein desalting of SbSGT1. A: Purification and analysis of the SbSGT1 protein using the desalting column; B: Analysis of desalted SbSGT1 by 12% SDS-PAGE. Lane 1–5 represents the SbSGT1 proteins eluted from the imidazole salt solution, and M represents the protein standard molecular weight.

## Appendix A

**Table A1:** Primer sequences.

Gene Name	Forward Primer Sequence
*SbSGT1*-qR	5′-GTAAACCTTGAATCGCCTGATG-3′
*SbSGT1*-qF	5′-TGAACTTGATCCTACGATGCAT-3′
*SbEIF4a*-qF	5′-AGGATTGGCACCAGAAGGGT-3′
*SbEIF4a*-qR	5′-CACATCAAGCCCCTTGCAGA-3′
*SbSGT1*-F	5′-GCGAATTCATGGCCGCGTCGGATCT-3′
*SbSGT1*-R	5′-CGCTCGAGTTAGTATTCCCACTTCTTGAGCT-3′
*NbEF1*-F	5′-AGAGGCCCTCAGACCAAC-3′
*NbEF1*-R	5′-TAGGTCCAAAGGTCACAA-3′

**Table A2:** Cis-acting elements of the *SbSGT1* promoter region.

Cis-Elements	Sequence(5′–3′)	Function	Number
MYC	CATGTG	Drought response	3
GC-motif	CCCCCG	Enhancer-like element involved in anoxic specific inducibility	1
GT1-motif	GGTTAA	Light responsive element	5
ABRE	ACGTG	Cis-acting element involved in the abscisic acid responsiveness	1
WUN-motif	TTATTACAT	Wound-responsive element	2
TGA-element	AACGAC	Auxin-responsive element	2
P-box	CCTTTTG	Gibberellin-response	1
TGACG-motif	TGACG	Cis-acting regulatory element involved in the MeJA-responsiveness	1
CAT-box	GCCACT	Cis-acting regulatory element related to meristem expression	1
CGTCA-motif	CGTCA	Cis-acting regulatory element involved in the MeJA-responsiveness	1
MRE	AACCTAA	MYB binding site involved in light response	1
GATA-motif	AAGATAAGATT	Part of a light responsive element	1
TATA-box	TATA, TATAAAA, TATAAA, TATAA, TAAAGATT, TATAAAT, TATATTTATATTT, TATATA, ATATAA, ATATAT	Core promoter element around -30 of transcription start	41
ARE	AAACCA	Cis-acting regulatory element essential for the anaerobic induction	1
