# Supplementary figures and images for: Expression Analysis Reveals That Sorghum Disease Resistance Protein SbSGT1 Is Regulated by Auxin
Source: Biology (Basel). 2022 Jan 2;11(1):67. doi: 10.3390/biology11010067 (PMC8772907; doi:10.3390/biology11010067)

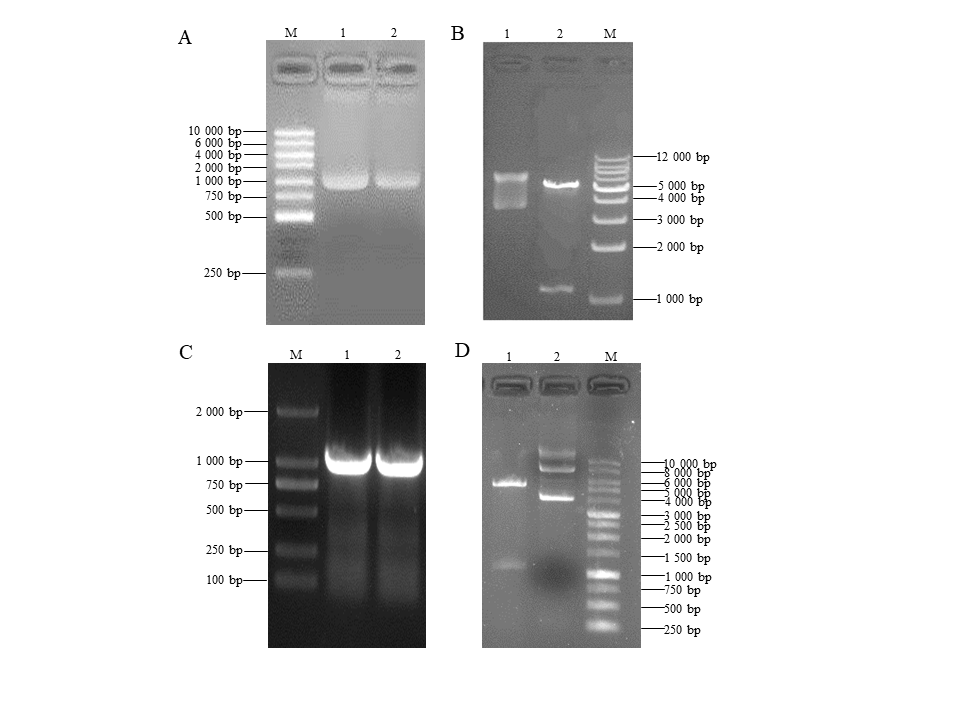

Supplement: Supplementary file 1 [file biology-11-00067-s001.zip › Figure S1.tif]

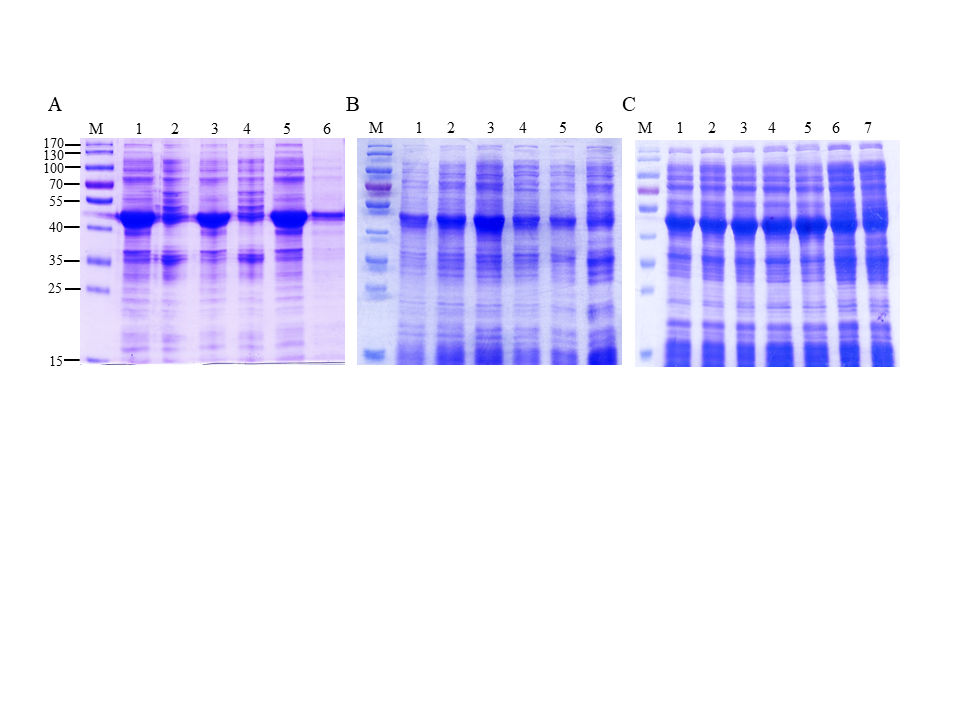

Supplement: Supplementary file 1 [file biology-11-00067-s001.zip › Figure S2.tif]

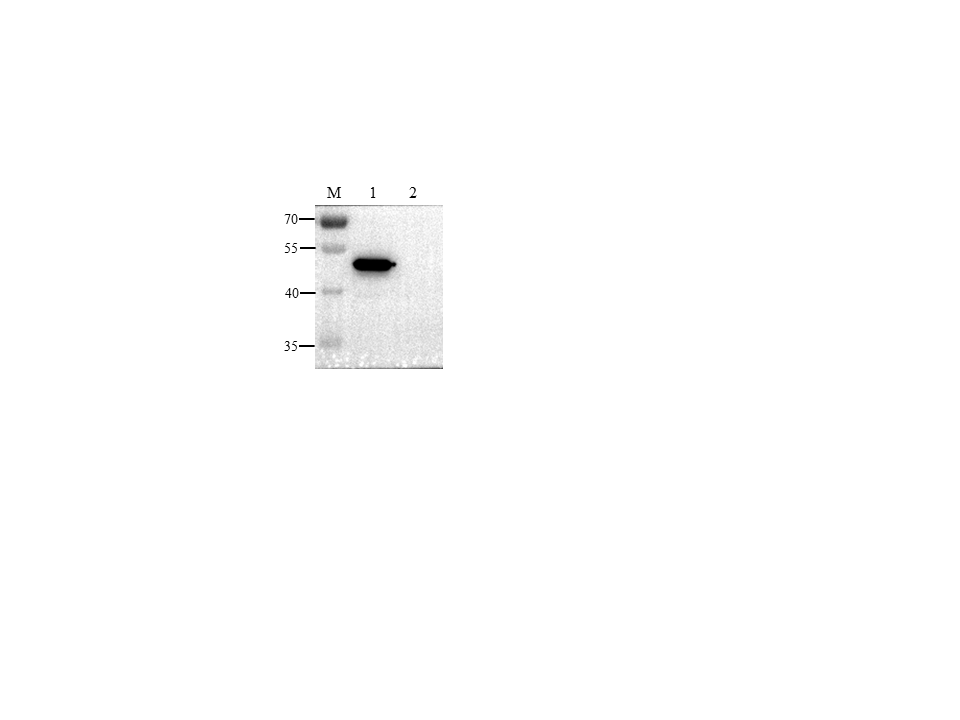

Supplement: Supplementary file 1 [file biology-11-00067-s001.zip › Figure S3.tif]

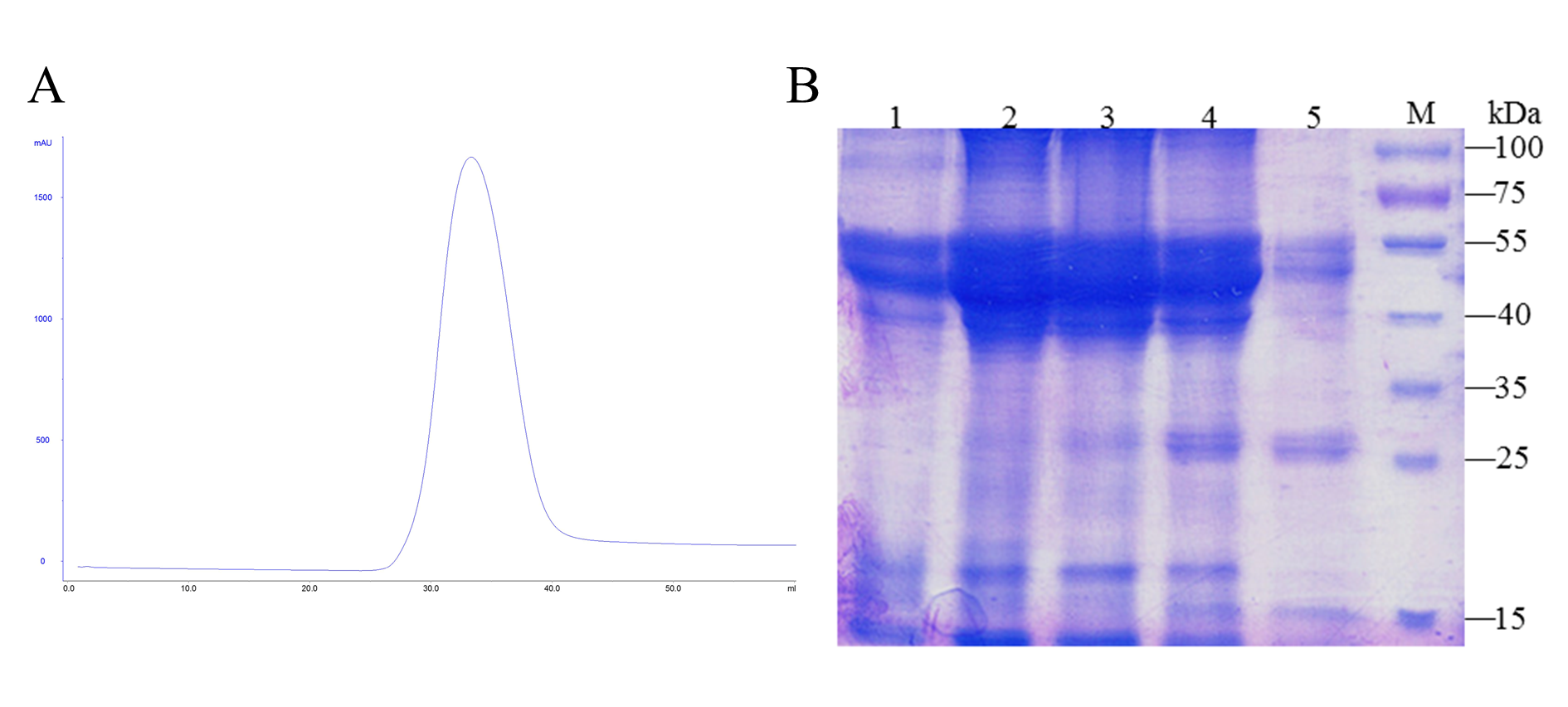

Supplement: Supplementary file 1 [file biology-11-00067-s001.zip › Figure S4.tif]

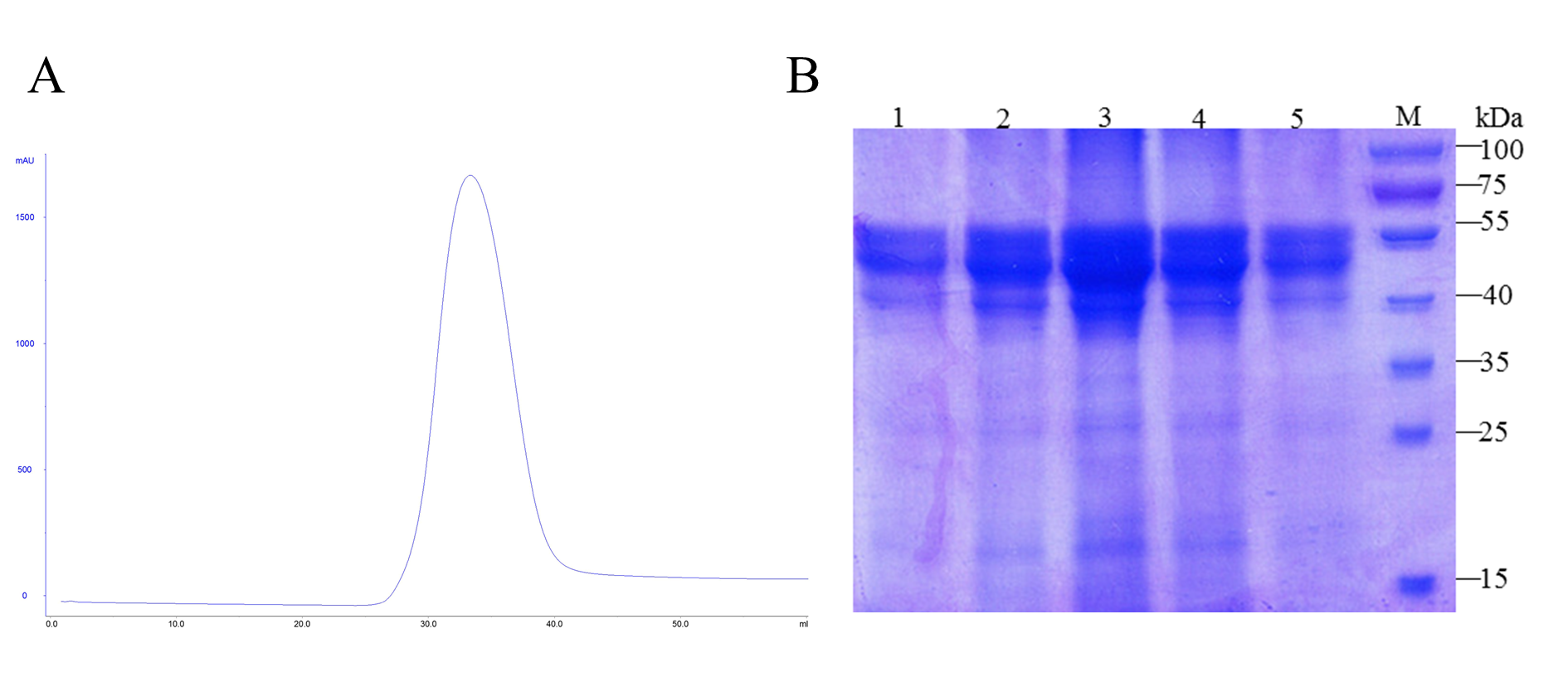

Supplement: Supplementary file 1 [file biology-11-00067-s001.zip › Figure S5.tif]
